# Which Cognitive Behavioral Therapy Formats Work for Depression in Dementia Family Caregivers ?

**DOI:** 10.1093/geroni/igab046.2471

**Published:** 2021-12-17

**Authors:** Yue Sun, Zhi-wen Wang

**Affiliations:** 1 School of Nursing, School of Nursing, Peking University, Beijing, China (People's Republic); 2 Peking University Health Science Center, Beijing, Beijing, China (People's Republic)

## Abstract

Cognitive behavioral therapy (CBT) has been shown to be effective to delay cognitive decline for family dementia caregivers (DCs). However, whether cognitive intervention could effectively reduce depression through internet, group, telephone, individual, unguided self-help and combined formats remains unclear. Pubmed, Embase, Cumulative Index to Nursing and Allied Health Literature (CINAHL), the Cochrane Central Register of Controlled Trials, Web of science, China National Knowledge Infrastructure database, Chinese Biomedical Literature database and Wan Fang database were systematically searched. A total of 34 studies were included in our analysis based on a series of rigorous screenings, which comprised 3577 DCs. We conducted a network meta-analysis (NMA) to evaluate the relative effects and rank probability of different CBT delivery formats. A series of analyses and assessments, such as the pairwise meta-analysis and the risk of bias, were performed concurrently. Compared with controls, internet, telephone, and individual showed the largest improvement on depressive symptoms, whereas the unguided self-help delivery format was less effective. Internet delivery formats had the highest probability among the five CBT delivery formats. Our study indicated that the internet might be the best delivery formats for reducing the depression of family DCs. The findings from our study may be useful for policy makers and service commissioners when they make choices among different CBT delivery formats.

